# Lactate is always the end product of glycolysis

**DOI:** 10.3389/fnins.2015.00022

**Published:** 2015-02-27

**Authors:** Matthew J. Rogatzki, Brian S. Ferguson, Matthew L. Goodwin, L. Bruce Gladden

**Affiliations:** ^1^Department of Health and Human Performance, University of Wisconsin-PlattevillePlatteville, WI, USA; ^2^Department of Biomedical Sciences, University of MissouriColumbia, MO, USA; ^3^Department of Orthopaedics, and Huntsman Cancer Institute, University of UtahSalt Lake City, UT, USA; ^4^School of Kinesiology, Auburn UniversityAuburn, AL, USA

**Keywords:** aerobic, anaerobic, lactate dehydrogenase, mitochondria, NADH, pyruvate, cytosolic lactate shuttle

## Abstract

Through much of the history of metabolism, lactate (La^−^) has been considered merely a dead-end waste product during periods of dysoxia. Congruently, the end product of glycolysis has been viewed dichotomously: pyruvate in the presence of adequate oxygenation, La^−^ in the absence of adequate oxygenation. In contrast, given the near-equilibrium nature of the lactate dehydrogenase (LDH) reaction and that LDH has a much higher activity than the putative regulatory enzymes of the glycolytic and oxidative pathways, we contend that La^−^ is always the end product of glycolysis. Cellular La^−^ accumulation, as opposed to flux, is dependent on (1) the rate of glycolysis, (2) oxidative enzyme activity, (3) cellular O_2_ level, and (4) the net rate of La^−^ transport into (influx) or out of (efflux) the cell. For intracellular metabolism, we reintroduce the Cytosol-to-Mitochondria Lactate Shuttle. Our proposition, analogous to the phosphocreatine shuttle, purports that pyruvate, NAD^+^, NADH, and La^−^ are held uniformly near equilibrium throughout the cell cytosol due to the high activity of LDH. La^−^ is always the end product of glycolysis and represents the primary diffusing species capable of spatially linking glycolysis to oxidative phosphorylation.

## Introduction

In the nineteenth century, ≈80 years after the discovery of lactate (La^−^) by Scheele (Kompanje et al., 2007), Louis Pasteur noticed that facultative yeast cells grew more under aerobic than anaerobic conditions, yet the consumption of sugar was decreased and fermentation to alcohol was less under aerobic conditions (Pasteur, 1861). Previously, Pasteur (1858) had recognized that some types of yeast fermented sugar to La^−^ under anaerobic, but not aerobic conditions. This phenomenon (for both alcohol and La^−^ fermentation) has been called the Pasteur Effect (Barnett and Entian, 2005). A parallel phenomenon was discovered in skeletal muscle and whole animals. For skeletal muscle Fletcher and Hopkins (1907) reported that La^−^ accrued in anaerobic frog muscles at rest. During stimulation, La^−^ concentration ([La^−^]) increased rapidly in anaerobic amphibian muscle, but disappeared when these fatigued muscles were allowed to recover in an oxygen (O_2_) rich environment. Subsequently, Meyerhof demonstrated conclusively that glycogen was the precursor of La^−^ in isolated muscles, and the full glycolytic pathway was elucidated by the early 1940s (Meyerhof, 1942; Brooks and Gladden, 2003). The traditional dogma was built upon this framework and other research on hypoxia: Pyruvate is the end product of glycolysis under aerobic conditions and La^−^ is the end product when O_2_ is insufficient. Schurr (2006) discussed this dogma from the viewpoint of brain metabolism.

It is widely accepted that intracellular PO_2_ values of ≈0.5 Torr or less result in O_2_-limited oxidative phosphorylation, a condition termed dysoxia (Connett et al., 1990), with ensuing La^−^ production and accumulation. However, Stainsby and Welch (1966) reported La^−^ efflux from an ostensibly well-oxygenated contracting muscle. Subsequently, Jöbsis and Stainsby (1968) observed La^−^ production and release from a contracting canine skeletal muscle while the NAD^+^/NADH redox couple was becoming more oxidized, an indication of adequate O_2_ supply. Using a different approach, myoglobin cryomicrospectroscopy, to determine PO_2_ in dog gracilis muscle contracting at progressively faster rates, Connett et al. (1986) found increasing La^−^ efflux without evidence of dysoxia; the lowest PO_2_ values were generally on the order of 2 Torr. Richardson et al. (1998) used proton magnetic resonance spectroscopy (MRS) to determine myoglobin saturation (and thereby intracellular PO_2_) in humans during graded exercise. In parallel experiments with the same type of exercise, La^−^ efflux was determined via arteriovenous concentration differences and blood flow. They found La^−^ efflux in the presence of intracellular PO_2_ levels (~3 Torr) that should not limit oxidative phosphorylation. Véga et al. (1998) also reported that isolated, stimulated nerve tissue releases lactate during aerobic conditions.

These findings, along with other abundant circumstantial evidence indicate that net La^−^ production and efflux from cells can occur under aerobic conditions (Gladden, 2004a,b). In fact, Brooks (2000) proposed that “lactate was produced all the time in fully oxygenated cells and tissues.” Schurr (2006) discussed this proposition in detail, proposing that “glycolysis always proceeds to its final step, the LDH reaction and the formation of lactate” in brain tissue but most likely in many other tissues as well. Subsequently, Schurr and Payne (2007) and Schurr and Gozal (2012) provided supportive experimental evidence for this postulate in hippocampal brain slices. Here, we embrace this concept, proposing that even in the absence of net La^−^ accumulation, and in the presence of plentiful O_2_, La^−^ is the natural end product of glycolysis. Importantly, we use basic biochemical principles to undergird this concept and re-introduce the Cytosol-to-Mitochondria Lactate Shuttle.

## The LDH reaction is a near equilibrium reaction

La^−^ is formed in the following reaction that is catalyzed by the enzyme lactate dehydrogenase (LDH):

$${\text{Pyruvate}}^{-}+\text{NADH}+{\text{H}}^{+}↔{\text{Lactate}}^{-}+{\text{NAD}}^{+}$$

The equilibrium constant is strongly in favor of La^−^ (1.62 × 10^11^ M^−1^) (Lambeth and Kushmerick, 2002), and LDH activity is high relative to the putative regulatory enzymes in the glycolytic pathway in skeletal muscle (Connett and Sahlin, 2011), liver, kidney, cardiac muscle, spleen, and fat (Shonk and Boxer, 1964), brain (Iwangoff et al., 1980; Morland et al., 2007), and both malignant and benign mammary tumors (Larner and Rutherford, 1978; Balinsky et al., 1984). Importantly, LDH activity is also high in comparison to the putative regulatory enzymes of pyruvate oxidation; see Spriet et al. (2000) for skeletal muscle, Morland et al. (2007) for brain, and Marie and Shinjo (2011) for brain cancer. While measures of tissue La^−^ to pyruvate ratios are scarce, some example values are ≈7:1 for liver (Liaw et al., 1985), ≈10–13:1 for resting skeletal muscle (Sahlin et al., 1976; Liaw et al., 1985), and values as high as 159:1 in skeletal muscle immediately following exhaustive dynamic exercise (Sahlin et al., 1976). Reference values for the La^−^ to pyruvate ratio in the brain, using microdialysis probes, average 23:1 (Reinstrup et al., 2000; Sahuquillo et al., 2014). Typically, the ratio rises following traumatic brain injury, even in the absence of ischemia or low tissue PO_2_ {≥ 25(Sahuquillo et al., 2014); ≥40 (Vespa et al., 2005)}. Despite standardization of techniques, microdialysis values do not necessarily reflect real tissue concentrations (Sahuquillo et al., 2014). Nevertheless, these La^−^ to pyruvate microdialysis values for human brain are not far afield from values (≈13:1) obtained on rat brain homogenates (Ponten et al., 1973). Overall, the high [La^−^] relative to [pyruvate] even with adequate O_2_ supply, reinforces the role of LDH activity in determining La^−^ appearance. The high LDH activity and La^−^-leaning equilibrium constant of the LDH reaction are key elements in the proposition that La^−^ is the major end product of glycolysis under essentially all metabolic conditions. Simply put, any time glycolysis is operative, regardless of local oxygen tension, La^−^ is being formed in most types of tissues. However, the amount of La^−^ produced and actually accumulated (i.e., an increased [La^−^]) can be altered by factors such as O_2_ tension, metabolic rate, available mitochondrial activity, and other factors.

## Fates of pyruvate

Potential fates of pyruvate are listed below. We propose that none of these processes occurs at a rate that matches the initial conversion of pyruvate to La^−^, thus ensuring that La^−^ is always the end product of glycolysis.

1. Efflux from the cell primarily via monocarboxylate transporters (MCTs). However, La^−^ is always present in a higher concentration than pyruvate and will depart cells at a faster rate than will pyruvate.
2. Conversion to alanine via the near equilibrium alanine aminotransferase reaction which has an equilibrium constant of about 1 (Tiidus et al., 2012), so alanine concentration should approximate pyruvate concentration and the conversion of pyruvate to alanine should not detract from the conversion of pyruvate to La^−^.
3. Gluconeogenic/Glyconeogenic reactions. In gluconeogenic tissues, pyruvate can be converted to oxaloacetate in a reaction catalyzed by pyruvate carboxylase (Pascoe and Gladden, 1996). In skeletal muscle glyconeogenesis, pyruvate can be converted to malate with catalysis by malic enzyme (Pascoe and Gladden, 1996) or more likely to phosphoenolpyruvate via reversal of the pyruvate kinase reaction (Donovan and Pagliassotti, 2000). These reactions represent “reversal” of glycolysis and they begin with La^−^, the natural end product of glycolysis. In the brain, glycogen is most abundant in astrocytes and sparse to negligible in neurons (Cataldo and Broadwell, 1986). Although pyruvate carboxylase is expressed in cultured astroglial cells, oligodendrocytes, microglial cells, and ependymocytes (Murin et al., 2009), we are unaware of any information on the ability of any of these cells to synthesize glycogen from La^−^.
4. Transport across the mitochondrial inner membrane with subsequent conversion to Acetyl-CoA via the pyruvate dehydrogenase (PDH) reaction followed by entry into the tricarboxylic acid cycle and oxidation. Pyruvate crosses the inner mitochondrial membrane via simple diffusion and facilitated diffusion; the transporters are an MCT (Hashimoto et al., 2006) and the mitochondrial pyruvate carrier (Divakaruni and Murphy, 2012). For ongoing oxidation of pyruvate, NADH shuttling into the mitochondrial matrix by the malate-aspartate and glycerol phosphate shuttles is equally important as pyruvate transport.

The constant presence of La^−^ and its accumulation during periods of glycolytic stimulation is evidence that the LDH reaction predominates over these alternative fates of pyruvate.

**Figure 1:**
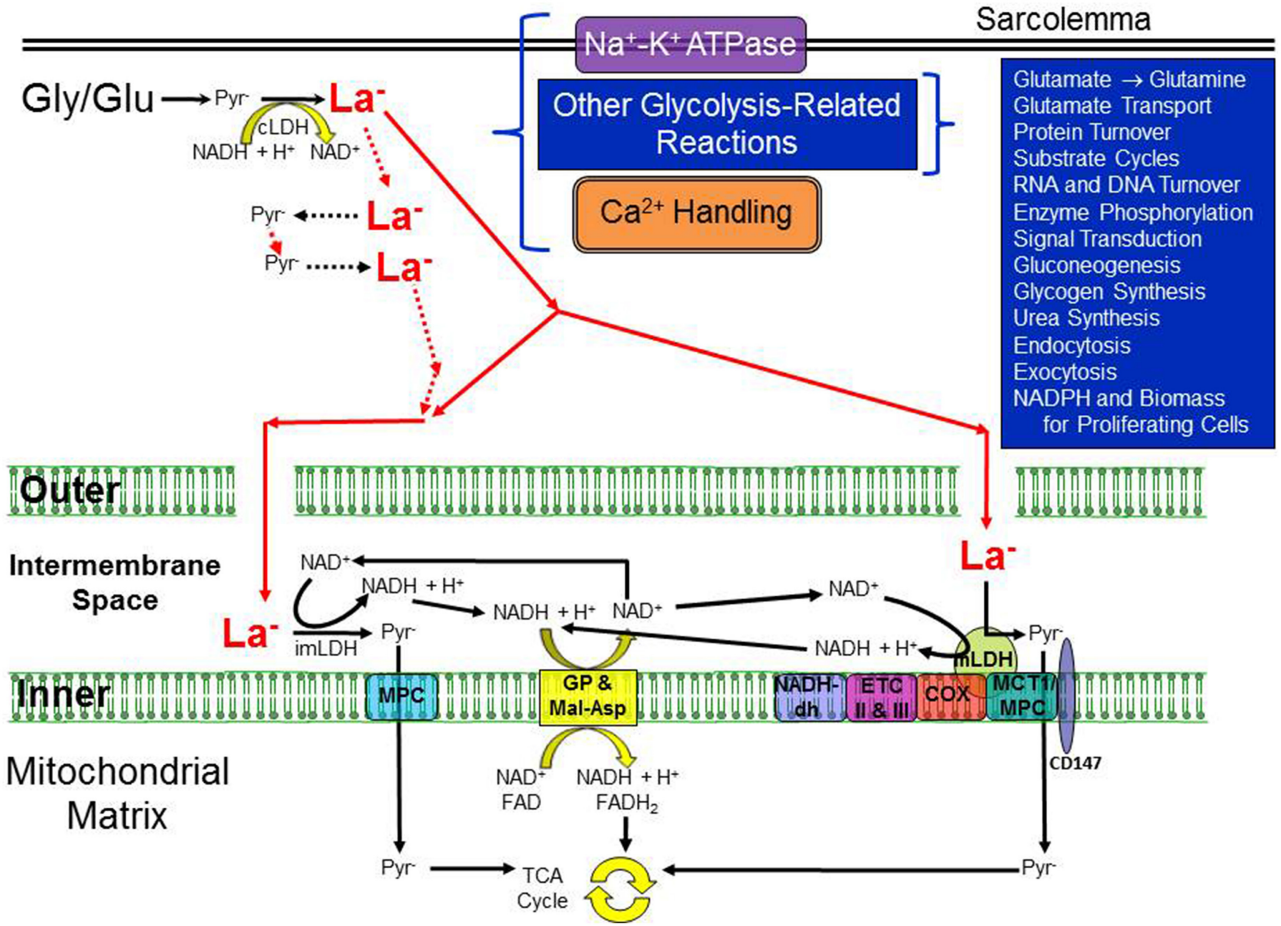
**Illustration of the essential elements of the re-introduced Cytosol-to-Mitochondria Lactate Shuttle**. A high activity of cytosolic LDH is considered to guarantee La^−^ formation in the cytosol under virtually all conditions but especially during periods of increased glycolytic activity. Not all cells would necessarily exhibit all of the processes shown in the upper right quadrant. La^−^ can be formed throughout the cytosol; two particular locations are noted for which there is evidence of compartmentation with glycolysis, one in association with the Na^+^-K^+^-ATPase pump in the sarcolemma and the other for skeletal and cardiac muscle, the Ca^2+^-ATPase in the sarcoplasmic reticulum membrane. The sarcolemma is illustrated by the thick double lines at the top of the cartoon whereas the inner and outer mitochondrial membranes are dramatically enlarged to demonstrate possible La^−^ pathways. The gaps in the outer mitochondrial membrane illustrate that it is freely permeable to most small molecules (but probably not permeable to LDH). La^−^ is shown in bold and red, and larger than pyruvate (Pyr^−^) to indicate that La^−^ is typically present in much higher concentration than Pyr^−^ (i.e., a high La^−^/Pyr^−^ ratio). Whether La^−^ is converted back to Pyr^−^ outside the intermembrane space, inside the space, or via a mitochondrial LDH, the resulting NADH + H^+^ would be shuttled across the inner mitochondrial membrane via the malate-aspartate and glycerol phosphate shuttles. Pyr^−^ could be transported across the inner mitochondrial membrane by either a mitochondrial pyruvate carrier (MPC) or a monocarboxylate transporter (MCT), both of which have been identified in the inner membrane. COX indicates cytochrome oxidase; cLDH, cytosolic lactate dehydrogenase; CD147, single-span transmembrane glycoprotein; ETC II and III, electron transport chain complexes II and III; Gly, glycogen; Glu, glucose; imLDH, LDH in the intermembrane space; Inner, inner mitochondrial membrane; La^−^, lactate; MCT1, monocarboxylate transporter 1; mLDH, mitochondrial LDH; MPC, mitochondrial pyruvate carrier; NADH-dh, NADH dehydrogenase complex I; Outer, outer mitochondrial membrane; Pyr^−^, pyruvate. Conceived from (1) Stainsby and Brooks (1990), (2) Hashimoto et al. (2006), and (3) Gladden (2008).

Figure 1 illustrates a model of intracellular metabolism which we call the “Cytosol-to-Mitochondria Lactate Shuttle”; its origin can be traced to a review of La^−^ metabolism by Stainsby and Brooks (1990). Because of the high LDH activity and an equilibrium constant far in the direction of La^−^, La^−^ is always the predominant result of glycolysis. However, formation of La^−^ is not synonymous with La^−^ accumulation and increased [La^−^]. Mitochondria constitute a sink for pyruvate and under conditions of slow glycolytic activity with ample O_2_, oxidation in most cells is sufficient to closely match production by glycolysis; transmembrane La^−^ flux will vary between slow release and slow uptake with release being the more typical condition. In a manner analogous to creatine kinase and the Phosphocreatine Shuttle, LDH holds pyruvate and La^−^ in equilibrium throughout the cell cytosol. In this scenario, La^−^ is the primary species that travels to the neighborhood of the mitochondrial reticulum, most likely to the intermembrane space where LDH is attached to the outer side of the inner mitochondrial membrane (Hashimoto et al., 2006; Gladden, 2008). Here, La^−^ is converted to pyruvate for entry into the mitochondria, given the relative “sink” for pyruvate. Simultaneously, NADH is regenerated from the reversal of the LDH reaction and its pair of electrons is shuttled across the inner mitochondrial membrane by the malate-aspartate and glycerol phosphate shuttles. An important difference from the Phosphocreatine Shuttle is that two key components, La^−^ and pyruvate, unlike phosphocreatine, can cross the plasma membrane and leave the cell.

The Cytosol-to-Mitochondria paradigm posits that La^−^ is always formed during glycolysis, even if La^−^ is not accumulating and [La^−^] is stable. Of course, if O_2_ is so low that oxidative phosphorylation is inhibited, then La^−^ production will exceed the rate at which oxidative metabolism can use pyruvate and NADH, causing [La^−^] and La^−^ efflux to rise. Also, if glycolytic activity increases even with ample O_2_ levels, as in skeletal muscle contracting at a moderate intensity or perhaps in activated astrocytes (Pellerin and Magistretti, 2011), La^−^ production will not be matched by pyruvate oxidation and [La^−^] will rise as will transport of La^−^ out of the cell. Similarly, if glycolytic enzyme activity is enhanced and/or mitochondrial function (oxidative enzyme activity) is downregulated such that glycolysis is favored over oxidation, there will be an ongoing mismatch between La^−^ production and subsequent pyruvate and NADH oxidation resulting in elevated [La^−^] and La^−^ efflux. This latter situation is observed in “Warburg” cancer cells (Semenza, 2008) and in COPD patients during whole body exercise *in vivo* (Maltais et al., 1996).

With endurance exercise training, skeletal muscle mitochondrial content is increased (Holloszy and Coyle, 1984), and there is now a larger sink for pyruvate. Increased mitochondrial oxidative activity requires lower levels of stimulators (e.g., ADP) for a particular oxidative phosphorylation rate; these same stimuli are allosteric stimulators of key glycolytic enzymes so glycolysis is reduced. Additionally, if La^−^ membrane transport is inhibited, particularly in cells that already have a mismatch in which glycolysis is favored over oxidative metabolism, it is likely that cellular [La^−^] will rise with potentially deleterious effects on the cell (Le Floch et al., 2011). Further, strong inhibition of total LDH activity in glycolytic cells should prevent equilibrium and thereby reduce La^−^ production, accumulation, and efflux (Fantin et al., 2006). However, the effect of changing the LDH isozyme pattern independent of inhibition or reduction of total LDH activity is still yet to be fully resolved (Downer et al., 2006).

## Future directions: influence of LDH isoform and applications to tumor metabolism

What impact does LDH isoform have and how might this knowledge be applied to the treatment of diseases with altered metabolism, like cancers?

First, LDH is a tetrameric enzyme composed of two protein subunits which total approximately 135 kDa (Cahn et al., 1962). The tetramer can assemble as five separate isozymes by forming all combinations of the M (muscle) form (product of the Ldh-A gene) or the H (heart) form (product of the Ldh-B gene) producing: M_4_ (= A_4_ = LDH5), M_3_H_1_ (= A_3_B_1_ = LDH_4_), M_2_H_2_ (= A_2_B_2_ = LDH3), M_1_H_3_ (= A_1_B_3_ = LDH2), and H_4_ (= B_4_ = LDH1). Results from investigations *in vitro* indicate differing kinetic properties with respect to substrate affinity and inhibition among these isozymes. The M-dominated isozymes have 3.5–7 times higher K_m_-values for pyruvate and La^−^ than the H-dominated forms. Further, the H_4_ types are inhibited by pyruvate at concentrations above ~0.2 mM while the M_4_ types are little affected by pyruvate concentrations as high as 5 mM (Plagemann et al., 1960; Stambaugh and Post, 1966; Quistorff and Grunnet, 2011b). The H_4_ isozyme is inhibited by [La^−^] above 20–40 mM while the M_4_ isozyme is less inhibited by high [La^−^] (Stambaugh and Post, 1966). These points have been offered as evidence for functional differences in cellular metabolism of various tissues with the heart forms promoting oxidation while the muscle forms facilitate formation of La^−^ (Cahn et al., 1962). The LDH isozyme distribution found in nature fits with these characteristics determined *in vitro*. For example, fast-twitch, glycolytic, type II skeletal muscle fibers have a greater proportion of M-type LDH isozyme whereas slow-twitch, oxidative, type I skeletal muscles as well as cardiac muscle have a greater proportion of the H-type LDH isozyme (Van Hall, 2000). Congruently, endurance exercise training decreases the proportion of the M-type LDH isozyme in the trained muscles (Van Hall, 2000). In the brain, astrocytes (which are postulated to have a higher glycolytic metabolism), have a greater proportion of the M-type LDH isozyme, whereas neurons (which are asserted to have a higher oxidative metabolism), have a greater proportion of the H-type LDH isozyme (Schurr, 2006; Pellerin and Magistretti, 2011). In tumors, glycolytic “Warburg-type” cells have a greater proportion of M-type LDH isozyme while more oxidative cancer cells have a greater proportion of H-type LDH isozyme (Semenza, 2008). So, the circumstantial evidence of LDH isozyme distribution patterns coincides with the perceived function of the LDH isozymes as determined *in vitro*.

The evidence cited above has led to the conclusion that LDH isozyme pattern is a causative factor in La^−^ metabolism. To further elucidate the role of LDH isozyme apportionment as a coordinator of La^−^ metabolism, Summermatter et al. (2013) undertook an investigation to test the role of peroxisome proliferator-activated receptor-γ coactivator 1α (PGC-1α) as a regulator of LDH isozyme subtype expression. PGC-1α is known to be important in the coordination of cellular energy metabolism (Wu et al., 1999). In response to a variety of stimuli, PGC-1α stimulates mitochondrial biogenesis, promotes transition of skeletal muscle to a more oxidative phenotype, and contributes to altered carbohydrate and lipid metabolism (Liang and Ward, 2006).

Summermatter et al. (2013) studied muscle-specific PGC-1α transgenic mice as well as muscle-specific PGC-1α knockout mice and found (1) lower blood [La^−^] in the transgenic animals, and higher blood [La^−^] in the knockout animals in response to endurance exercise, and (2) reduced expression of M-type LDH in the transgenic animals and reduced H-type LDH in the knockout animals. These authors concluded, as their title asserts, that “skeletal muscle PGC-1 α controls whole-body La^−^ homeostasis through estrogen-related receptor α-dependent activation of LDH B and repression of LDH A.” In their view, the LDH isozyme pattern is a major player in whole body metabolism of La^−^.

However, there are under-appreciated admonitions regarding LDH isozyme functions and their potential roles in metabolism. First, the aforementioned kinetic properties for LDH isoforms were determined *in vitro* at 20 or 25°C, and the K_m_-values for pyruvate increase with temperature, approximately doubling at 37°C compared to 25°C (Latner et al., 1966; Quistorff and Grunnet, 2011b). Previously, Newsholme and Leech (1983), Van Hall (2000), Newsholme (2004), Gladden (2008), and Quistorff and Grunnet (2011a), have raised significant questions about the role of LDH isozyme profiles in La^−^ production vs. utilization, noting that: (1) enzymes do not change the equilibrium constant of a reaction; (2) the LDH reaction is near equilibrium, minimizing allosteric effects; (3) differences in LDH isozyme function *in vivo* are possibly quite small because of the higher physiological temperatures and binding to structures or other proteins; (4) the concentrations of La^−^ and pyruvate needed for LDH inhibition *in vitro* are much higher than the highest concentrations observed *in vivo*; and (5) LDH inhibition *in vitro* may be due to traces of the enol form of pyruvate that are less likely to be present *in vivo*.

Although Summermatter et al. (2013) state with conviction that LDH isoform pattern is a major factor in whole body La^−^ metabolism, there is a fatal flaw in their design. They ignored the fact that PGC-1 α transgenic mice have increased mitochondrial proliferation and oxidative phosphorylation enzymes, whereas PGC-1α knockout mice have significant reductions in cytochrome oxidase and citrate synthase activities (Arany et al., 2005). In our opinion, these changes in mitochondrial function, the previously noted high total LDH activity irrespective of isozyme pattern, and the near equilibrium nature of this reaction render the conclusions of Summermatter et al. (2013) untenable. Therefore, we conclude that the exact physiological and biochemical roles of LDH isozymes *in vivo* remain to be definitively elucidated.

Finally, with regard to tumor metabolism, understanding that La^−^ is the end product of glycolysis is paramount to designing interventions for targeting cancers. Briefly, experiments by Cori and Cori (1925) and by Warburg et al. (1927) showed that tumors appeared to be avidly consuming glucose and producing La^−^. Subsequent dogma in tumor metabolism has held that tumors exhibit a “Warburg Effect,” producing and exporting La^−^. However, we now know that not only do different tumor types handle La^−^ differently (some are net producers; some are net consumers), but even within a single tumor there may be shuttling between different cell types; a cell to cell La^−^ shuttle (Semenza, 2008). Many cancer cells are poor consumers of lactate (Sonveaux et al., 2008) sparking speculation that a La^−^-protected hypoglycemia may be therapeutic (Nijsten and van Dam, 2009). In contrast, some tumors avidly use La^−^ as a fuel, and respond to supplemental La^−^ with increased proliferation and vascularity, likely a direct result of upregulation of vascular endothelial growth factor (VEGF) and hypoxia-inducible factor 1α (HIF-1α). In a recent study on an animal model of a sarcoma, Goodwin et al. (2014) reported that La^−^ drove sarcomagenesis in the absence of hypoxia. Amazingly, our understanding of La^−^ metabolism in cancer remains unsettled almost 90 years after Warburg's first studies.

## Conclusions

Our understanding of La^−^ formation has changed drastically since its discovery. Traditionally, pyruvate has been thought to be the end product of glycolysis when O_2_ is present and La^−^ the end product during periods of dysoxia. In the late twentieth century and early twenty-first century it was discovered that O_2_ is not limiting to oxidative phosphorylation under most cellular conditions, and La^−^ is indeed produced even when there is no limitation on the rate of O_2_ delivery to mitochondria. Further reflection on the activity of the LDH enzyme and the equilibrium constant of its reaction advance the proposition that La^−^ is the primary end product of glycolysis under most, if not all metabolic conditions in most cells. The role of the different LDH isozymes in metabolism is not as clearly evident as most researchers suggest, and we conclude that their exact function remains undiscovered. Whether or not we are correct about the Cytosol-to-Mitochondria Lactate Shuttle as described here and the uncertain role of the LDH isoforms will be difficult to evaluate under conditions *in vivo*. One approach is modeling *in silico*. Understanding the exact mechanisms of glycolysis and La^−^ metabolism will not only deepen our understanding of metabolism in healthy tissues, but will also lend insight into diseased or injured tissues, with the most obvious applications being the deranged carbohydrate metabolism present in cancer cells (Vander Heiden et al., 2009) and cerebral metabolism following traumatic brain injury (Brooks and Martin, 2014).

### Conflict of interest statement

The authors declare that the research was conducted in the absence of any commercial or financial relationships that could be construed as a potential conflict of interest.
